# Importance-Weighted Locally Adaptive Prototype Extraction Network for Few-Shot Detection

**DOI:** 10.3390/s25195945

**Published:** 2025-09-23

**Authors:** Haibin Wang, Yong Tao, Zhou Zhou, Yue Wang, Xu Fan, Xiangjun Wang

**Affiliations:** 1 School of Computer Science and Technology, Anhui University of Technology, Maanshan 243032, China; whb1553432@outlook.com (H.W.); taoyong@ahut.edu.cn (Y.T.); 13407934921@163.com (Z.Z.); 2 Anhui Province Key Laboratory of Digital Twin Technology in Metallurgical Industry, Anhui University of Technology, Maanshan 243032, China; 3 State Key Laboratory of Precision Measuring Technology and Instruments (Tianjin University), Tianjin 300072, China; tjuxjw@126.com

**Keywords:** few-shot object detection, local adaptive prototype, imbalanced diversity sampling, non-linear fusion, mean average precision

## Abstract

Few-Shot Object Detection (FSOD) aims to identify new object categories with a limited amount of labeled data, which holds broad application prospects in real-life scenarios. Previous approaches usually ignore attention to critical information, which leads to the generation of low-quality prototypes and suboptimal performance in few-shot scenarios. To overcome the defect, an improved FSOD network is proposed in this paper, which mimics the human visual attention mechanism by emphasizing areas that are semantically important and rich in spatial information. Specifically, an Importance-Weighted Local Adaptive Prototype module is first introduced, which highlights key local features of support samples, and more expressive class prototypes are generated by assigning greater weights to salient regions so that generalization ability is effectively enhanced under few-shot settings. Secondly, an Imbalanced Diversity Sampling module is utilized to select diverse and challenging negative sample prototypes, which enhances inter-class separability and reduces confusion among visually similar categories. Moreover, a Weighted Non-Linear Fusion module is designed to integrate various forms of feature interaction. The contributions of the feature interactions are modulated by learnable importance weights, which improve the effect of feature fusion. Extensive experiments on PASCAL VOC and MS COCO benchmarks validate the effectiveness of our method. The experimental results reflect the fact that the mean average precision from our method is improved by 2.84% on the PASCAL VOC dataset compared with Fine-Grained Prototypes Distillation (FPD), and the AP from our method surpasses the recent FPD baseline by 0.8% and 1.8% on the MS COCO dataset, respectively.

## 1. Introduction

Recent breakthroughs in deep neural networks have enabled significant progress across a broad spectrum of vision tasks [1]. However, the performance of such models typically depends on large-scale annotated datasets. The collection of datasets is often labor-intensive and time-consuming, which restricts the scalability and practical deployment of vision systems [2]. In contrast, the human visual system is capable of quickly learning to recognize new objects from only a few examples—an ability that current deep learning models still lack. This limitation has driven the development of Few-Shot Object Detection (FSOD), which aims to narrow the gap between machine perception and human learning. In FSOD, knowledge from base classes is transferred to novel categories, allowing new objects to be recognized using only a small number of labeled examples [3].

Owing to its practical significance in recognizing new object types under minimal supervision, FSOD has attracted surging interest [4]. In contrast to few-shot image classification, FSOD presents a greater challenge as it requires both accurate classification and precise localization under extremely limited training data. To overcome the challenge, most existing methods are designed under either a meta-learning paradigm or a transfer-learning paradigm. Despite promising advances, two fundamental challenges remain unresolved.

Firstly, many existing methods rely on fixed, global prototypes constructed via average pooling over support features. Although this strategy is straightforward, it often produces low-quality prototypes that fail to capture discriminative local cues. For example, Meta Region-Based Convolutional Neural Network (Meta R-CNN) and Few-Shot Object Detection via Feature Reweighting (FSRW) [5] both adopt global prototype representations, but their performance degrades notably in cases with high intra-class variation or partial occlusion since critical object parts cannot be emphasized. Moreover, the issue of category confusion remains a fundamental bottleneck. Few-Shot Object Detection via Contrastive Proposal Encoding (FSCE) [6] explicitly points out that global prototypes often lead to overlapping feature distributions for semantically similar categories (e.g., cow vs. sheep), which severely limits inter-class separability. Similarly, Decoupled Faster Region-Based Convolutional Neural Network (DeFRCN) [7] demonstrates that the learned prototypes are prone to bias toward base categories without decoupling feature reuse and classification, which further aggravates misclassification on novel categories. Beyond this, fixed prototypes are inherently static and query-agnostic, which means that they cannot adapt to instance-specific or context-dependent variations. The argument is also discussed in Information-Coupled Prototype Elaboration (ICPE) [8] and Query Adaptive Few-Shot Object Detection (QA-FewDet) [9], which attempts but only partially succeeds in addressing this rigidity. Collectively, these limitations represent the key weaknesses of prior FSOD approaches and significantly hinder their generalization to novel categories.

**Figure 1:**
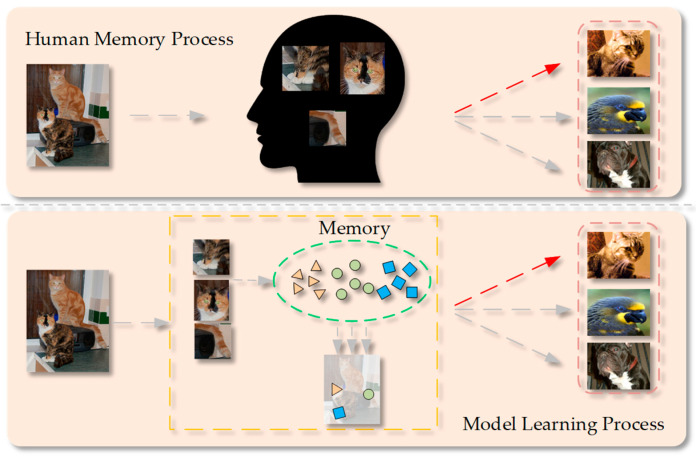
Summary of model ideas.

To synthesize higher-quality prototypes from support sets, an Importance-Weighted Local Adaptive Prototype (IWLAP) attention mechanism is proposed to accentuate key local features, which mimics how humans remember new objects through their distinctive local features. The conceptual diagram is shown in Figure 1. Moreover, the IWLAP module can extract salient features to serve as additional prototypes in candidate selection, thus enriching the prototype pool.

Previous prototype sampling methods have also been reviewed. Early approaches, such as Meta R-CNN, rely on class-specific aggregation where prototypes within the same class are merged and inter-class distinctions are largely ignored. In contrast, Class-Agnostic Aggregation [10] is proposed to capture shared intra-class variance across different categories, which improves generalization performance by encouraging the learning of inter-class differences and reducing bias toward base classes. In this work, the ability of the model to distinguish between similar categories is further enhanced, and Hard negative sample prototypes that are visually similar to positive features are selectively introduced, as well as easy negatives that are clearly distinct. Therefore, the model is guided to better separate confusing categories through the contrastive sampling strategy.

Moreover, it is recommended to incorporate learnable weights into the feature aggregation module, enabling the model to better manage feature fusion. Extensive experiments on the PASCAL VOC and MS COCO datasets validate the effectiveness of our method. The key contributions of this paper are shown as follows.

- The representation of support features is enhanced by extracting and integrating critical information from support feature maps via the IWLAP module.
- An Imbalanced Diversity Sampling (IDS) strategy and a Weighted Non-Linear Fusion (W-NLF) module are introduced to improve the feature fusion capability of the FSOD network.
- Our method achieves remarkable performance improvements across multiple experiments, delivering superior results on two prominent FSOD benchmarks.

## 2. Related Work

### 2.1. Task Definition

In accordance with the established few-shot object detection paradigm, a dataset *D* is partitioned into two distinct category sets, namely base classes *C_base_* and novel classes *C_novel_*. The base classes *C_base_* contain ample training samples, whereas each novel class in *C_novel_* is limited to precisely *K* annotated instances. The objective of FSOD is to develop a detector trained on *D*, which can recognize objects from both *C_base_* and *C_novel_* concurrently. Crucially, the base and novel classes are mutually exclusive, which satisfies *C_base_* ∩ *C_novel_* = Ø.

### 2.2. General Object Detection

Significant progress has been achieved for general object detection in recent years, which is largely driven by the development of deep convolutional neural networks (CNNs). With the advancement of CNNs, both detection accuracy and computational efficiency are enhanced across various visual tasks.

Two-stage detectors, such as Faster Region-based Convolutional Neural Network [11], remain among the widely adopted approaches. In these methods, region proposals are first generated by a Region Proposal Network (RPN). Then, classification and bounding box regression are performed on these proposals in a second stage. This two-step pipeline enables precise localization and classification, thereby achieving high detection accuracy. One-stage detectors, including You Only Look Once and Single Shot MultiBox Detector [12], have also been utilized. In these models, object categories and bounding boxes are predicted in a single forward pass. Faster inference speed can be achieved, although accuracy may be slightly reduced, especially for small or overlapping objects. Several improvements have been introduced to enhance detection across different object scales and spatial variations. Feature Pyramid Networks [13] are used to fuse multi-scale features through a top-down structure with lateral connections, which helps improve the detection of small objects. Deformable Convolutions [14] introduce flexible sampling over spatial locations, allowing the model to better adapt to object shapes and deformations. Transformer-based models have also been explored for object detection. The Meta Detection Transformer (Meta-DETR) [15] reformulates object detection as a set prediction problem, in which self-attention mechanisms are used to model global context, and handcrafted components such as anchor boxes and non-maximum suppression are removed.

Despite these advances, general object detection methods strongly rely on large-scale annotated datasets, where each category is trained with abundant labeled samples. The training and testing categories are assumed to be the same, and the models are optimized mainly for accuracy within these categories. As a result, their performance often drops sharply when training samples are scarce, which is especially true for rare categories or long-tailed distributions. To address this limitation, FSOD has been proposed. FSOD fundamentally differs from general detection in that it does not assume abundant annotations for all categories but rather focuses on adapting to novel categories with only a handful of annotated instances.

### 2.3. Few-Shot Object Detection

FSOD aims to detect novel object categories by only using a few annotated instances per class, which is particularly relevant in real-world scenarios where data collection and manual labeling are costly or impractical [16]. Unlike general object detection, where models are trained and tested on the same set of categories with sufficient annotations, FSOD explicitly separates base classes (well-annotated) and novel classes (data-scarce). Knowledge must be transferred from base classes to novel classes, which makes the task inherently more challenging.

Currently, there are three mainstream paradigms in FSOD, including the transfer-learning-based paradigm, the data augmentation-based paradigm, and the Meta-learning-based paradigm [17]. In the transfer learning paradigm, a two-stage training strategy is typically adopted. In the base training stage, a detector is trained in base classes with abundant labeled samples to learn general-purpose representations. During the fine-tuning phase, the detector adapts to new classes by only using a few labeled instances, typically updating only the classification and regression heads while keeping the backbone network frozen, such as in the two-stage fine-tuning method (TFA) [18]. The Towards Stabilized Few-Shot Object Detection [19] approach enhances performance stability and reduces forgetting through a simple sample normalization technique. FSCE further enhances the standard fine-tuning pipeline by introducing a contrastive proposal encoding branch, which learns discriminative Region of Interest (RoI) features by enforcing intra-class compactness and inter-class separability. Other variants, such as Multi-scale Positive Sample Refinement (MPSR) [20], incorporate class-balancing strategies, feature re-weighting to address distribution imbalance and semantic confusion.

Data augmentation-based methods constitute an effective solution to the inherent data scarcity in FSOD, aiming to enrich both the diversity and quantity of training samples at either the image or feature level. Such augmentation enhances the generalization ability of detectors in novel classes. The core idea is to simulate intra-class variance and augment limited support data through various strategies. At the feature level, methods like Dual-Awareness Attention [21] and DeFRCN perturb or recombine deep features while preserving semantic integrity to increase robustness. Another promising direction involves hallucinating pseudo support samples by using generative networks or transformation modules, as seen in approaches like Hallucination [22]. These pseudo samples are designed to mimic real intra-class variations and provide stronger supervision for learning discriminative prototypes.

In addition to transfer learning and data augmentation, meta-learning is an important paradigm in FSOD. The meta-learning paradigm aims to learn how to learn from limited data by simulating few-shot tasks during training [23]. Following the episodic training framework, the model is trained on episodes consisting of support and query sets. The support set provides a few annotated samples for each class, and the query set is used for evaluation [24]. This episodic strategy enables the model to quickly adapt to novel classes with limited supervision. A key component of meta-learning methods is the use of prototypes, which are typically constructed by averaging the support features of the same class. The classification of query objects is then performed based on the similarity between query features and these class prototypes by using metric functions such as cosine similarity or Euclidean distance. Early methods, such as Meta R-CNN and FSRW, lay the foundation by incorporating prototype-guided RoI feature enhancement. More recent works, such as ICPE and QA-FewDet, improve prototype quality by introducing query-aware representation and dynamic prototype generation.

## 3. Our Approach

**Figure 2:**
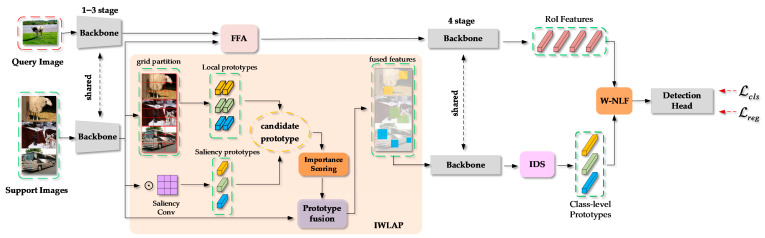
The overall framework of the proposed network for FSOD tasks.

Figure 2 shows the overall framework of the proposed FSOD network. Firstly, the low-level features of the query image and support images are, respectively, extracted by using the first three stages of the ResNet-101 backbone network. Secondly, the subsequent process is divided into two branches.

In the query branch, the query and support features are fed into the Fine-grained Feature Aggregation (FFA) module. This module extracts fine-grained prototypes from the support feature map and densely matches them to the query feature map so that an enhanced query representation enriched with support information is obtained. It should be noted that the FFA module is derived from Fine-grained Prototypes Distillation (FPD). Then, the RoI features of the query image are extracted by using the fourth stage of the ResNet-101 backbone network.

In the support branch, the support features are first divided into local feature regions through a fixed grid partitioning. These local regions, together with the support feature map, are fed into the IWLAP module to generate candidate prototypes, which consist of both local prototypes and importance-aware prototypes. The candidate prototypes are then evaluated based on their importance scores, and the selected prototypes are used for support feature fusion. The fused support features are further processed by the fourth stage of ResNet-101 to extract class-level prototypes. Subsequently, the IDS strategy selects a subset of these prototypes, which are integrated with the RoI features through the W-NLF module to obtain the final fused representation. This fused representation is eventually utilized for the downstream classification and regression tasks.

### 3.1. Importance-Weighted Locally Adaptive Prototype

**Figure 3:**
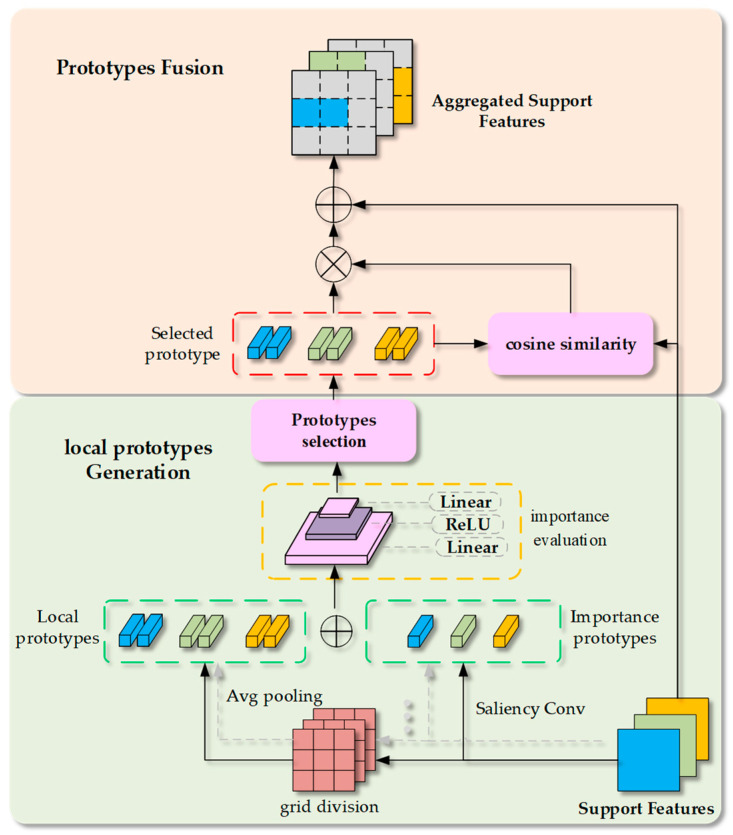
The architecture of the IWLAP extraction module.

An importance-weighted locally adaptive prototype extraction module (as shown in Figure 3) is introduced to capture representative and discriminative cues effectively from the limited support samples. In this module, a set of local prototypes is dynamically selected from the support feature maps, which are designed to summarize the salient local parts of the support objects and provide enhanced semantic guidance for subsequent fusion processes.

#### 3.1.1. Candidate Prototypes Acquisition

Given a support feature map $F\in R^{B\times C\times H\times W}$, a coarse partition of the spatial structure is first obtained by using grid-based pooling. Specifically, the support feature map is partitioned into *N_g_* grids, and each grid is pooled to produce a local patch-level prototype $p_{i}^{\left(g\right)}$, namely

(1) $$p_{i}^{\left(g\right)}=AvgPool(F_{i}^{\left(g\right)}),$$

where $F_{i}^{\left(g\right)}$ denotes the spatial region corresponding to the *i*-th grid cell.

In parallel, we compute an importance-aware scoring map by using a convolutional filter and extract the top-*K* most salient positions $p_{i}^{\left(t\right)}$ by selecting the top-*K* responses across the flattened spatial dimension, as shown in Equation (2).

(2) $$p_{i}^{\left(t\right)}=TopK(Conv(F)),$$

Ultimately, the candidate prototype set *P_cand_* is obtained through concatenating the grid prototypes and the importance prototypes, namely

(3) $$P_{cand}=\left[p_{i}^{\left(g\right)},\ldots ,p_{N_{g}}^{\left(g\right)},p_{i}^{\left(t\right)},\ldots ,p_{K}^{\left(t\right)}\right],$$

#### 3.1.2. Prototypes Importance Evaluation and Selection

An importance rating network ϕ is employed to quantify the informativeness of each candidate prototype. This network consists of two fully connected layers followed by a ReLU activation, and produces an importance score *w_i_* for each candidate prototype $p_{i}^{\left(c\right)}$:

(4) $$w_{i}=\phi (p_{i}^{\left(c\right)}),\;\forall p_{i}^{\left(c\right)}\in P_{cand},$$

where $w_{i}$ reflects the contribution of prototype $p_{i}^{\left(c\right)}$ to the detection task.

The local prototypes are selected according to the importance scores, in which two selection strategies (i.e., weighted sampling and diversity-aware selection) are utilized.

For the weighted sampling strategy, the selection probability of each candidate prototype is determined by the corresponding importance score $w_{i}$, which reflects the semantic relevance and representativeness of that prototype with respect to the target object. The softmax function is applied across all candidate prototypes to normalize the raw importance scores into a valid probability distribution. Given a set of importance scores $\left\{w_{1},w_{2},\ldots ,w_{m}\right\}$, the selection probability $p_{i}$ of the *i*-th prototype is computed by

(5) $$p_{i}=\frac{\exp(w_{i})}{\sum_{j=1}^{m}\exp(w_{j})},$$

where *m* denotes the number of candidate prototypes.

Then, stochastic sampling is performed to select *N* prototypes from the probability distribution. The above probabilistic mechanism allows a degree of diversity to be preserved in the selected prototypes while still favoring more informative regions.

For the diversity-aware selection strategy, the prototype with the highest importance score is first selected from the candidate set to compose the selected prototype set *P_sel_*. For subsequent selections, the cosine similarity between remaining candidate prototypes $p_{r}^{\left(c\right)}$ and the selected prototypes $p_{i}^{\left(s\right)}$ is used to evaluate the diversity. The hyperparameter λ controls the emphasis on diversity between candidate prototypes and the already selected set. A larger λ encourages the model to prioritize diversity, making it more suitable for scenarios with large intra-class variations. In contrast, a smaller λ biases the model toward selecting highly important prototypes, even if they are similar to those already chosen. By tuning λ, a balance between importance and diversity can be flexibly achieved during prototype selection, enabling adaptation to different task requirements. The diversity-enhanced score ${\tilde{w}}_{i}$ for each remaining candidate prototype can be calculated by

(6) $$\begin{array}{l} {\tilde{w}}_{i}=w_{i}+\lambda \cdot \min\left(1-\cos\left(p_{r}^{\left(c\right)},p_{i}^{\left(s\right)}\right)\right)\\ r=1,\ldots ,m-len(P_{sel}),\;\forall p_{i}^{\left(s\right)}\in P_{sel}, \end{array},$$

where *len*(*) is used to calculate the size of the selected prototype set.

Then, the candidate prototype with the maximum enhancement score is selected and added to the set of selected prototypes, which is repeated until a total of *N* prototypes is selected.

Herein, it must be emphasized that the weighted sampling strategy is adopted on the Pascal VOC dataset in the following experimental trials, and the diversity-aware selection strategy is adopted on the MS COCO dataset. We further investigate the impact of different prototype selection strategies on datasets of varying complexity. On the PASCAL VOC dataset, the weighted sampling strategy is adopted to yield the best performance. This can be attributed to the relatively small number of object categories (20 classes) and the limited intra-class variations. In such scenarios, detection performance benefits more from emphasizing the most salient and discriminative local regions, while excessive pursuit of diversity may introduce redundant or noisy prototypes that do not contribute additional information. In contrast, the diversity-aware selection strategy proves more effective on the MS COCO dataset, which contains a substantially larger number of categories (80 classes) and exhibits higher intra-class diversity and inter-class similarity. By explicitly encouraging the selection of semantically distinct prototypes, this approach enhances spatial and semantic coverage of the support features. Such diverse prototype representations are particularly important for distinguishing between visually similar categories (e.g., “dog” vs. “cat” or “chair” vs. “couch”) and handling complex backgrounds and occlusions.

#### 3.1.3. Prototypes Fusion

To inject the extracted prototypes back into the support features and enhance the semantic capacity, a prototype fusion module inspired by attention mechanisms is designed. Firstly, the support feature is flattened into *F_s_*, and the cosine similarity *A_i,s_* between each spatial location features $f_{s}$ and the prototype is computed by

(7) $$A_{i,s}=\frac{f_{s}\cdot p_{i}^{\left(s\right)}}{\left\|f_{s}\right\|\cdot \left\|p_{i}^{\left(s\right)}\right\|},\;\forall s\in HW,\;i\in N,$$

where *N* is the total number of samples to be selected, and *H* and *W* denote the height and width dimensions of the support feature map *F_s_*.

Secondly, the prototype attention weights are obtained after the cosine similarity scores *A_i,s_* are processed through softmax, which are used for weighted reconstruction of the features. Eventually, the residual fusion techniques are applied to integrate the prototypes and inject them back into the support features.

The weighted prototype selection mechanism offers a robust and adaptive strategy for prototype selection in few-shot object detection, which balances salience and diversity, and enables the model to focus on the most representative features for detection tasks.

### 3.2. Imbalanced Diversity Sampling

The aggregation of RoI features and class-level prototype features is regarded as a key step in meta-learning-based FSOD. Meta R-CNN utilizes a simple class-specific aggregation method, which only aggregates RoI features with prototypes of the same class. Variational Feature Aggregation (VFA) adopts a completely random class-agnostic selection approach, which randomly selects a prototype from one category for RoI feature fusion. FPD argues that pure randomness can impair performance and proposes a balanced class-agnostic sampling (B-CAS) [25], which selects a pair of positive and negative prototypes to aggregate with RoI features in parallel. In view of that, a more refined hierarchical negative sampling strategy (i.e., IDS strategy) is employed in our work. The employed IDS strategy aims to select a subset of support sample prototypes that include both representative positive sample prototypes and negative sample prototypes with diverse semantic similarity to the query class. Specifically, hard and easy negative sample prototypes are distinguished based on the semantic distance of the prototypes to the target class. The negative sample prototypes are sorted by semantic similarity, and only the prototypes within a small range at the beginning and end of the sequence are used as hard and easy negative sample prototypes. The selection of semantically similar hard negative prototypes enables greater focus to be placed on challenging distinctions, thereby enhancing the discriminative ability of the learned features.

#### 3.2.1. Positive Prototype Construction

Let us define the query class tags as *y^+^*, and the set of support sample prototypes *P* whose labels match *y^+^* is first identified. We randomly select *N_p_* positive examples from the prototype set *P*, and construct a class prototype *v^+^* by averaging the semantic vectors:

(8) $$v^{+}=\frac{1}{N_{p}}\sum v_{i},$$

where $v_{i}$ denotes the semantic feature vector of the *i*-th support sample.

The semantic feature vector *v*^+^ is considered the semantic centroid of the positive class, which provides a stable reference for measuring inter-class similarity.

#### 3.2.2. Negative Sample Sampling

For the negative sample prototypes *v*^−^ whose label does not equal *y*^+^, the semantic distance from the negative sample prototypes to the positive prototype is calculated by

(9) $$d_{i}=1-\cos(v_{i}^{-},v^{+}),$$

where cos(*,*) denotes cosine similarity.

A lower distance *d_i_* implies greater semantic resemblance to the positive class, thereby identifying a hard negative sample that may cause confusion during training.

To balance hard and easy negative sample prototypes, a hyperparameter *α*∈(0, 1) is introduced to control the proportion of hard negatives among all negative sample prototypes, as shown in Equation (10). As a result, a sufficient number of hard negatives are involved in training, which prevents the model from being dominated by easy negatives and enhances its ability to distinguish between visually similar but semantically different objects.

(10) $$N_{hard}=\max(1,\left[\alpha \cdot T_{n}\right]),N_{easy}=T_{n}-N_{hard},$$

where *T_n_* is the total number of negative sample prototypes, *N_easy_* and *N_hard_* are, respectively, the total number of easily negative sample prototypes and hard negative sample prototypes.

Then, all negative sample prototypes are sorted according to the semantic distance *d_i_*, and the top *ηN_hard_* negative sample prototypes are considered as candidates for hard negatives and the bottom *ηN_easy_* negative sample prototypes are considered as candidates for easy negatives. Here, *η* represents an adjustable factor indicating the random sampling range, which determines the size of the random selection scope for hard and easy negative sample prototypes. The value of *η* should not be too large, as it may blur the distinction between hard and easy negative sample prototypes. It is recommended to set the value of *η* within the range of 2 to 3. As a result, *N_hard_* and *N_easy_* sample prototypes are, respectively, randomly selected, and the selected support sample prototypes are fused with RoI features in a ratio of *P_sample_*:*N_hard_*:*N_easy_* = 1:1:1.

The negative sample prototypes are refined by using semantic distance to ensure the provision of rich semantic information, which encourages the model to learn more robust and discriminative representations under limited supervision.

### 3.3. Weighted Non-Linear Fusion

W-NLF enhances the Non-Linear Fusion (NLF) process by introducing a weighting mechanism, which allows the importance of different features to be adjusted dynamically when RoI features and support prototype features are combined. Therefore, more representative fused features can be obtained.

To adjust the contributions of different interaction features, each interaction type (i.e., addition *agg_add_*, subtraction *agg_sub_*, concatenation *agg_cat_*) is assigned a dynamic weight, which is managed by a trainable parameter *w_fusion_* and non-linearly transformed via a ReLU activation function. The weighted interaction features are then concatenated with the original query features *q* and the fused features *agg* are eventually obtained after passing through a linear layer, as shown in Equation (11).

(11) $$agg=Linear(\left[\left[agg_{add},agg_{sub},agg_{cat}\right]\times w_{fusion},q\right])$$

The W-NLF module combines three feature interaction methods with dynamic weighting. The query and support features are allowed to be effectively merged, which improves performance in few-shot object detection.

## 4. Experiment and Results

### 4.1. Implementation Details

The experiments are conducted on a workstation running Ubuntu 20.04 LTS, which is equipped with an Intel^®^ Xeon^®^ Silver 4314 CPU @ 2.40 GHz and 512 GB of system RAM (Intel Corporation, Santa Clara, CA, USA). The training is performed on a single NVIDIA GeForce RTX 3090 GPU with 40 GB of memory (Nvidia Corporation, Santa Clara, CA, USA). All models are implemented in Python 3.8.20 using PyTorch 1.12.1 and accelerated by CUDA 11.3.

In this paper, our method is evaluated on two prominent FSOD benchmarks, namely PASCAL VOC [26] and MS COCO [27]. Following the experimental setup of reference [18] (Wang et al., 2020), identical class partitions and few-shot examples are utilized for experimental comparison.

For the PASCAL VOC dataset, the 20 classes are divided into 15 base and 5 novel classes under three different class partitions. Training is conducted on the VOC07 and VOC12 training/validation sets, and evaluation is performed on the VOC07 test set. Results are reported in terms of mean average precision (mAP) at IoU = 0.5 (AP50, mAP at 0.5 IoU threshold) for *K* = {1, 2, 3, 5, 10} shot settings.

For the MS COCO dataset, the 20 PASCAL VOC classes are considered as novel classes, and the remaining 60 classes are considered as base classes. A total of 5 k images from the COCO 2017 validation set are used for evaluation purposes, and the rest of the images in the COCO dataset are used for training. Results are reported in terms of average precision (AP) at IoU thresholds varying from 0.5 to 0.95 under the shot settings of *K* = {10, 30}.

The proposed method is implemented by using the MMdetection framework [28]. Specifically, a ResNet-101 backbone that has been pretrained on the ImageNet dataset is utilized, and single-scale feature maps are used for feature extraction. The query images are resized to a maximum dimension of 1333 pixels × 800 pixels during preprocessing, and the cropped instances from support images are scaled to 224 pixels × 224 pixels.

Training is conducted on an NVIDIA 3090 GPU with a total batch size of 4, and the Stochastic Gradient Descent optimizer is employed. In the base training phase, the proposed model is trained on the VOC and COCO datasets for 30 k and 220 k iterations, respectively. The initial learning rate is, respectively, set to 0.001 and 0.004, and the learning rate is adjusted to 0.001 during fine-tuning. Consistent with FPD, the identical loss functions are adopted to ensure comparability and consistency in the training process.

### 4.2. Main Results

In Table 1, both the single-run results and the average results from multiple runs for PASCAL VOC are presented. Seen from Table 1, our approach significantly outperforms other existing methods. In terms of average results over multiple runs, our approach achieves superior performance across all settings. Specifically, our method achieves an average improvement of 2.84% compared to the results of FPD. In the best single-run results, our method also exceeds previous approaches in several cases. Based on the average results of multiple runs, it can be observed that our method performs better when *K*-shot is small. However, the improvement of our method gradually slows down with the increase of *K*-shot, and the result does not surpass that of FPD in Novel sets 2–3 with *K* = {10} shots. Our framework is mainly designed to address information scarcity in low-shot cases. As the number of shots increases to 10, the raw features already contain sufficient information, which reduces the gain of our method. In addition, as the original features increase, the number of local prototypes increases. On this occasion, the local prototype distribution may be more dispersed, and the difficulty of aggregation increases, which affects the performance of our method.

We evaluate the proposed method on the MS COCO benchmark under two standard few-shot settings (i.e., 10-shot and 30-shot) and follow the common protocols in previous works. Table 2 shows the AP across two paradigms, namely the finetuning-based method and the meta-learning-based method. In the fine-tuning paradigm, DeFRCN achieves the best performance, and the AP, respectively, reaches 18.5% and 22.6% in the 10-shot and 30-shot settings, which can be attributed to its decoupled design of feature reuse and classification. In addition, the AP from method such as Few-Shot Object Detection via Association and Discrimination (FADI) reaches 12.2% and 16.1% under the two settings. Moreover, the AP from FSCE, respectively, achieves 11.9% and 16.4%, which demonstrates notable gains over the earlier baseline TFA, the performance of which remains at 9.1% and 12.1%. These results highlight the effectiveness of contrastive learning and feature balancing in low-data regimes. In the meta-learning paradigm, the AP from the proposed IWLAP, respectively, achieves 16.7% and 21.0% in the 10-shot and 30-shot settings, outperforming all other meta-based methods. In particular, the proposed IWLAP surpasses the recent strong baseline FPD, in which the AP is improved by 0.8% and 1.7% under the two settings, respectively. Compared with earlier approaches such as FSRW and Meta R-CNN, the improvement is even more substantial. These consistent gains validate the ability of IWLAP and IDS to capture fine-grained semantics under limited supervision. Overall, the extensive results have substantiated the superiority of our approach within the challenging MS COCO environment.

### 4.3. Ablation Study

To validate the effectiveness of each proposed component, we conduct a series of ablation studies on the PASCAL VOC dataset under {1, 3, 5}-shot settings. We progressively replace or enhance the baseline FPD framework by introducing our proposed modules, namely IDS, W-NLF, and IWLAP. Table 3 shows the results of the ablation study under AP50.

In Table 3, a significant improvement of 3.9% under the 1-shot setting is achieved when replacing the NLF of FPD with our W-NLF module. This is mainly because the W-NLF method can adjust the contribution of different interactive features, suppress noise, and produce a more expressive fusion representation. Moreover, performance is further enhanced by incorporating IDS, which indicates that introducing diverse negative sample prototypes effectively strengthens the model’s ability to distinguish between confusing categories. The IWLAP module can dynamically extract local prototypes. Even if only a few supporting images are available, the module will highlight the most discriminative object parts. This reduces the scarcity of information and provides richer guidance for query branches. The result from our method outperforms that of the original FPD by 8.9%, 3.7%, and 2.1% under the {1, 3, 5}-shot settings, respectively. The above experimental results clearly demonstrate the complementary benefits of the proposed modules.

**Figure 4:**
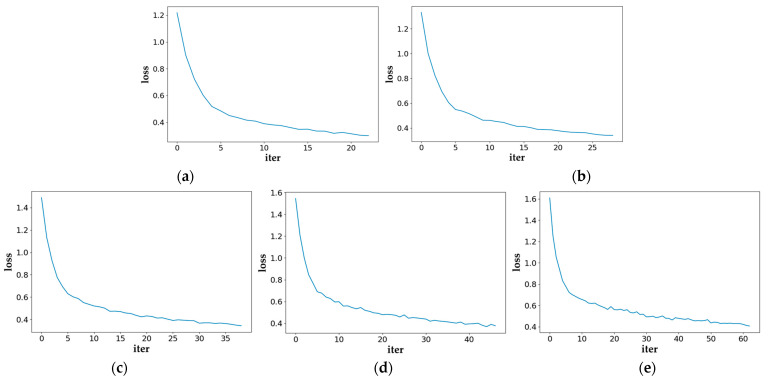
The loss of our method. (**a**) 1-shot (**b**) 2-shot (**c**) 3-shot (**d**) 5-shot (**e**) 10-shot.

Figure 4 illustrates the training-loss curves of the model on Novel Set 1 of the PASCAL VOC dataset across all shot settings. It can be seen from the figure that as the number of iterations increases, the loss curve tends to be stable, indicating that the model training process is relatively stable.

**Figure 5:**
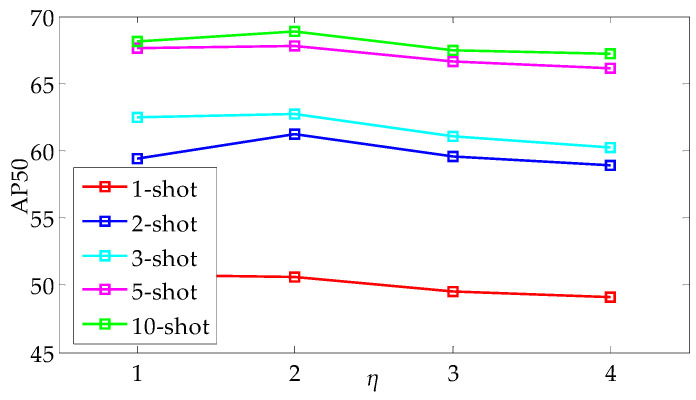
Ablation study on the interval control parameter *η*.

In addition, the ablation study on the interval control parameter *η* of sample selection is performed on {3, 5, 10}-shot settings. The performance is evaluated by using AP50, and Figure 5 shows the best results from multiple experimental trials. In Figure 5, the AP50 reaches the maximum when the parameter *η* is set to 2 across all shot settings. Therefore, the interval size of 2 is suggested to be optimal for sample selection on this occasion. The values of AP50 decrease as the interval size moves away from 2, which indicates a diminishing return on performance with larger or smaller interval sizes.

### 4.4. Visualize Detection Results

**Figure 6:**
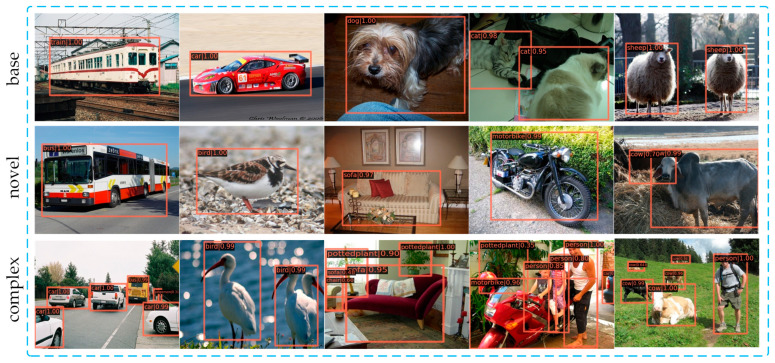
Visualization of detection results.

Figure 6 shows the detection results from our method. Specifically, the proposed model is trained on novel set 1 of the VOC dataset under the 10-shot setting and then evaluated on the VOC07 test set. The results in Figure 6 are divided into three partitions, namely base, novel, and complex. The base partition represents the detection of base classes, and the novel partition represents the detection of new classes. The complex partition represents the detection in images containing both base and new classes, or images with multiple new classes. The visualization results demonstrate that both base and novel classes can be effectively detected. In the complex partition, the majority of targets can be accurately detected, while there may exist a few cases of false detection. For instance, one sofa object is mistakenly detected as a chair class in the third image of the complex partition. However, the overall performance indicates that our method has great potential and effectiveness.

### 4.5. Computational Cost

Table 4 presents a comparison of model complexity and inference speed between the baseline FPD and our proposed IWLAP method on the PASCAL VOC and MS COCO datasets. The evaluation is conducted on a single NVIDIA 3090 GPU with a batch size of 1. From the perspective of the number of parameters, the method proposed in this paper only introduces a slight increase, indicating that the increased module does not have a large overhead on the model size. A similar trend can also be observed in FLOPs, where the computational cost rises by approximately 6.5% and 5.2% on VOC and COCO datasets, respectively.

Although the computational cost increases slightly, the inference speed remains almost unchanged. This is primarily because our method introduces only a few lightweight operations. Table 5 presents the network layers that contribute the most to the computational cost in our method. For example, average pooling is performed within each grid cell, a simple 3 × 3 convolution is applied to the support feature maps, and the importance scoring network ϕ together with cosine similarity computation, is influenced by the number of candidate prototypes, thereby leading to limited additional cost. The IDS module also introduces only a minor increase in computation. In summary, these observations indicate that the proposed method significantly enhances feature representation capability while maintaining nearly the same inference efficiency as the baseline.

## 5. Discussion

According to the above experimental results, our method achieves competitive results across multiple experimental benchmarks. For instance, the average performance over multiple runs surpasses the baseline by 8.9% on the Novel Set 1 of the VOC dataset in the 1-shot setting. On the PASCAL VOC dataset, the weighted sampling proves effective by emphasizing the most discriminative regions, while the diversity-aware strategy yields better results on the MS COCO dataset due to higher intra-class variation and inter-class similarity. This adaptability shows that our framework is not tuned for a single dataset but can dynamically adjust to different data distributions. Table 4 also indicates that the proposed modules only marginally increase the model complexity while maintaining inference speed nearly equivalent to the baseline. Nevertheless, the single-run results in Table 1 indicate that the upper bound of our approach remains marginally below the desired level.

One potential reason for this limitation is that the proposed model still relies on the conventional fixed-grid partitioning to define local regions, which may cause critical object parts to be incompletely captured. A feasible way to overcome the limitations of the fixed grid strategy is to use adaptive meshing. The core idea is to allocate partition regions dynamically based on object-aware cues, ensuring that critical parts of an object are better captured. Technically, such an approach is well supported by existing components in object detection. For instance, RPN has been shown to be effective in identifying discriminative object regions. This information can be used to guide adaptive partitioning by refining cell boundaries toward regions with higher semantic importance. Furthermore, deformable convolution and attention mechanisms in transformer-based detectors demonstrate that adaptive receptive fields can be learned in an end-to-end manner, providing empirical evidence that partitioning can also be optimized dynamically. By integrating these mature techniques, adaptive grid partitioning is technically achievable and has the potential to generate more representative local prototypes, thereby improving the discriminative capability of few-shot detectors in data-scarce scenarios.

Moreover, our work currently focuses on increasing the diversity of negative sample prototypes, while the influence of positive–negative sample imbalance is neglected. Therefore, improving the positive-sample sampling strategy will be a central direction for future research.

## 6. Conclusions

FSOD aims to detect novel categories with only a few annotated instances, and significant challenges exist because of data scarcity and overfitting. Although meta-learning-based approaches have shown promise by leveraging prior knowledge to generalize across categories, they often struggle to fully utilize fine-grained local information and maintain the balance between prototype representation and feature diversity. In this paper, an improved FSOD framework incorporating three key components (i.e., IWLAP, IDS, and W-NLF) is proposed. Firstly, an IWLAP module is introduced to extract representative local prototypes from support features, and then the extracted local prototypes are integrated with the raw features, thereby enhancing class-specific information. Secondly, the IDS strategy is designed to improve negative sample selection by introducing diversity-aware sampling, which encourages the model to learn from more informative contrasts. Thirdly, the W-NLF module adaptively balances the contributions of different prototype fusion strategies during the feature integration process so that the robustness and expressiveness of the fused features are improved. Extensive experiments on standard benchmarks demonstrate that our approach significantly improves detection performance under novel settings, highlighting its effectiveness and potential in advancing FSOD research.

Our method enhances few-shot prototype learning, but there is still room for improvement. Currently, local prototype features are extracted via fixed grid partitioning of support feature maps, which is simple but sensitive to grid size. In addition, important semantic regions may be split across grid cells. The multi-resolution grid partitioning can be explored to capture both coarse and fine-grained patterns hierarchically in future work.

## Figures and Tables

**Table 1:** FSOD results (AP50) on the three splits of Pascal VOC dataset.

Method/Shot	Novel Set 1					Novel Set 2					Novel Set 3				
	1	2	3	5	10	1	2	3	5	10	1	2	3	5	10
Single run results:															
FSRW (Kang et al., 2019) [5]	14.8	15.5	26.7	33.9	47.2	15.7	15.3	22.7	30.1	40.5	21.3	25.6	28.4	42.8	45.9
Meta R-CNN (Yan et al., 2019) [3]	19.9	25.5	35.0	45.7	51.5	10.4	19.4	29.6	34.8	45.4	14.3	18.2	27.5	41.2	48.1
TFA w/cos (Wang et al., 2020) [18]	39.8	36.1	44.7	55.7	56.0	23.5	26.9	34.1	35.1	39.1	30.8	34.8	42.8	49.5	49.8
MPSR (Wu et al., 2020) [20]	41.7	42.5	51.4	55.2	61.8	24.4	29.3	39.2	39.9	47.8	35.6	41.8	42.3	48.0	49.7
Retentive (Fan et al., 2021) [29]	42.4	45.8	45.9	53.7	56.1	21.7	27.8	35.2	37.0	40.3	30.2	37.6	43.0	49.7	50.1
FSCE (Sun et al., 2021) [6]	44.2	43.8	51.4	61.9	63.4	27.3	29.5	43.5	44.2	50.2	37.2	41.9	47.5	54.6	58.5
Meta FR-CNN (Han et al., 2022a) [23]	43.0	54.5	60.6	66.1	65.4	27.7	35.5	46.1	47.8	51.4	40.6	46.4	53.4	59.9	58.6
**Meta-DETR (Zhang et al., 2022) [15]**	40.6	51.4	58.0	59.2	63.6	**37.0**	36.6	43.7	49.1	**54.6**	41.6	45.9	52.7	58.9	60.6
FCT (Han et al., 2022b) [30]	49.9	57.1	57.9	63.2	67.1	27.6	34.5	43.7	49.2	51.2	39.5	54.7	52.3	57.0	58.7
FPD (Wang et al., 2024) [25]	48.1	**62.2**	**64.0**	67.6	68.4	29.8	43.2	**47.7**	52.0	53.9	44.9	53.8	**58.1**	**61.6**	62.9
IWLAP (Our)	**50.6**	61.2	62.7	**67.8**	**68.9**	31.0	**43.7**	46.7	**53.7**	54.2	**45.1**	**54.0**	54.3	60.1	**63.0**
Average results over multiple runs:															
FSDetView (Xiao et al., 2022) [31]	24.2	35.3	42.2	49.1	57.4	21.6	24.6	31.9	37.0	45.7	21.2	30.0	37.2	43.8	49.6
DCNet (Hu et al., 2021) [32]	33.9	37.4	43.7	51.1	59.6	23.2	24.8	30.6	36.7	46.6	32.3	34.9	39.7	42.6	50.7
Meta-DETR (Zhang et al., 2022) [15]	35.1	49.0	53.2	57.4	62.0	27.9	32.3	38.4	43.2	51.8	34.9	41.8	47.1	54.1	58.2
DeFRCN (Qiao et al., 2021) [7]	40.2	53.6	58.2	63.6	66.5	29.5	39.7	43.4	48.1	52.8	35.0	38.3	52.9	57.7	60.8
FCT (Han et al., 2022b) [30]	38.5	49.6	53.5	59.8	64.3	25.9	34.2	40.1	44.9	47.4	34.7	43.9	49.3	53.1	56.3
FPD (Wang et al., 2024) [25]	41.5	52.8	58.4	64.9	67.1	28.2	38.7	43.8	50.3	**53.6**	34.9	48.6	**54.0**	58.4	**61.5**
IWLAP (Ours)	**50.4**	**60.3**	**62.1**	**67.0**	**68.5**	**30.2**	**43.4**	**46.5**	**50.4**	53.3	**44.0**	**50.4**	52.9	**59.5**	60.4

**Table 2:** FSOD results (AP) on the two splits of MS COCO dataset.

Method/Shots	10	30
Fine-tuning		
MPSR (Wu et al., 2020) [20]	9.8	14.1
TFA w/cos (Wang et al., 2020) [18]	9.1	12.1
Retentive (Fan et al., 2021) [29]	10.5	13.8
FSOD-UP (Wu et al., 2021) [33]	11.0	15.6
SRR-FSD (Zhu et al., 2021) [34]	11.3	14.7
CGDP+FSCN (Li et al., 2021) [35]	11.3	15.1
FSCE (Sun et al., 2021) [6]	11.9	16.4
FADI (Cao et al., 2021) [36]	12.2	16.1
DeFRCN (Qiao et al., 2021) [7]	**18.5**	**22.6**
Meta-learning		
FSRW (Kang et al., 2019) [5]	5.6	9.1
Meta R-CNN (Yan et al., 2019) [3]	8.7	12.4
QA-FewDet (Han et al., 2021) [9]	11.6	16.5
FSDetView (Xiao et al., 2020) [31]	12.5	14.7
Meta FR-CNN (Han et al., 2022) [23]	12.7	16.6
DCNet (Hu et al., 2021) [32]	12.8	18.6
CME (Li et al., 2021) [37]	15.1	16.9
FPD (Wang et al., 2024) [25]	15.9	19.3
IWLAP (Ours)	**16.7**	**21.0**

**Table 3:** The results (AP50) of the ablation study.

Method	B-CAS	NLF	FFA	IDS	W_NLF	IWLAP	Shot		
							1	3	5
FPD	✓	✓	✓				41.5	58.4	64.9
Ours		✓	✓	✓			45.4	59.3	65.7
			✓	✓	✓		49.3	60.1	66.7
			✓	✓	✓	✓	50.4	62.1	67.0

**Table 4:** The computational cost of inference.

Dataset	Method	Params (MB)	FLOPs (GB)	FPS (img/s)
VOC (20 class)	FPD	65.68	818.10	14.8
	IWLAP(Ours)	67.26	871.32	14.7
COCO (80 class)	FPD	66.5	1309.50	6.5
	IWLAP(Our)	68.12	1377.20	6.2

**Table 5:** The network layers that provide the main amount of calculation in our method.

Module	Layer Name	Input Size	FLOPs
IWLAP	Ang pooling	(20, 1024, 7, 2)	2.8 × 10^6^
	Conv2	(20, 1024, 14, 14)	7.22 × 10^7^
	importance scoring network ϕ	(20, 15, 1024)	6.29 × 10^8^
IDS	Avg pooling	(20, 1024, 14, 14)	4.01 × 10^6^
	cosine similarity	(19, 1024), (1, 1024)	7.9 × 10^4^

## Data Availability

The data presented in this study are available on request from the corresponding author.

## References

[1] Redmon J., Divvala S., Girshick R., Farhadi A. You Only Look Once: Unified, Real-Time Object Detection. Proceedings of the IEEE Conference on Computer Vision and Pattern Recognition.

[2] Zhang S., Wang W., Wang Z., Li H., Li R., Zhang S. (2024). Extreme R-CNN: Few-shot object detection via sample synthesis and knowledge distillation. Sensors.

[3] Yan X., Chen Z., Xu A., Wang X., Liang X., Lin L. Meta r-cnn: Towards general solver for instance-level low-shot learning. Proceedings of the 2019 IEEE/CVF International Conference on Computer Vision (ICCV).

[4] Köhler M., Eisenbach M., Gross H.M. (2023). Few-shot object detection: A comprehensive survey. IEEE Trans. Neural Netw. Learn. Syst..

[5] Kang B., Liu Z., Wang X., Yu F., Feng J., Darrell T. Few-shot object detection via feature reweighting. Proceedings of the IEEE/CVF International Conference on Computer Vision.

[6] Sun B., Li B., Cai S., Yuan Y., Zhang C. Fsce: Few-shot object detection via contrastive proposal encoding. Proceedings of the 2021 IEEE/CVF Conference on Computer Vision and Pattern Recognition (CVPR).

[7] Qiao L., Zhao Y., Li Z., Qiu X., Wu J., Zhang C. DeFRCN: Decoupled Faster R-CNN for Few-Shot Object Detection. Proceedings of the 2021 IEEE/CVF International Conference on Computer Vision (ICCV).

[8] Lu X., Diao W., Mao Y., Li J., Wang P., Sun X., Fu K. Breaking immutable: Information-coupled prototype elaboration for few-shot object detection. Proceedings of the AAAI Conference on Artificial Intelligence.

[9] Han G., He Y., Huang S., Ma J., Chang S.F. Query adaptive few-shot object detection with heterogeneous graph convolutional networks. Proceedings of the IEEE/CVF International Conference on Computer Vision.

[10] Han J., Ren Y., Ding J., Yan K., Xia G.-S. (2023). Few-Shot Object Detection via Variational Feature Aggregation. Proc. AAAI Conf. Artif. Intell..

[11] Ren S., He K., Girshick R., Sun J. (2017). Faster R-CNN: Towards real-time object detection with region proposal networks. IEEE Trans. Pattern Anal. Mach. Intell..

[12] Liu W., Anguelov D., Erhan D., Szegedy C., Reed S., Fu C.-Y., Berg A.C. SSD: Single shot multibox detector. Proceedings of the European Conference on Computer Vision.

[13] Lin T.-Y., Doll’ar P., Girshick R., He K., Hariharan B., Belongie S. Feature pyramid networks for object detection. Proceedings of the 2017 IEEE Conference on Computer Vision and Pattern Recognition (CVPR).

[14] Dai J., Qi H., Xiong Y., Li Y., Zhang G., Hu H., Wei Y. Deformable convolutional networks. Proceedings of the IEEE International Conference on Computer Vision.

[15] Zhang G., Luo Z., Cui K., Lu S., Xing E.P. (2022). Meta-DETR: Image-level few-shot detection with inter-class correlation exploitation. IEEE Trans. Pattern Anal. Mach. Intell..

[16] Zhang X., Zhang Z. (2023). Research on a traffic sign recognition method under small sample conditions. Sensors.

[17] Huang G., Laradji I., Vazquez D., Lacoste-Julien S., Rodriguez P. (2022). A survey of self-supervised and few-shot object detection. IEEE Trans. Pattern Anal. Mach. Intell..

[18] Wang X., Huang T.E., Darrell T., Gonzalez J.E., Yu F. (2020). Frustratingly simple few-shot object detection. arXiv.

[19] Ren Y., Yang M., Han Y., Li W. (2024). Towards Stabilized Few-Shot Object Detection with Less Forgetting via Sample Normalization. Sensors.

[20] Wu J., Liu S., Huang D., Wang Y. (2020). Multi-scale positive sample refinement for few-shot object detection. Proceedings of the European Conference on Computer Vision.

[21] Chen T.I., Liu Y.C., Su H.T., Chang Y.C., Lin Y.H., Yeh J.F., Chen W.C., Hsu W.H. (2021). Dual-awareness attention for few-shot object detection. IEEE Trans. Multimed..

[22] Zhang W., Wang Y.X. Hallucination improves few-shot object detection. Proceedings of the IEEE/CVF Conference on Computer Vision and Pattern Recognition.

[23] Han G., Huang S., Ma J., He Y., Chang S.F. (2022). Meta faster r-cnn: Towards accurate few-shot object detection with attentive feature alignment. AAAI Conf. Artif. Intell..

[24] Zhang T., Sun R., Wan Y., Zhang F., Wei J. (2023). Msffal: Few-shot object detection via multi-scale feature fusion and attentive learning. Sensors.

[25] Wang Z., Yang B., Yue H., Ma Z. (2024). Fine-grained prototypes distillation for few-shot object detection. AAAI Conf. Artif. Intell..

[26] Everingham M., Van Gool L., Williams C.K., Winn J., Zisserman A. (2010). The pascal visual object classes (voc) challenge. Int. J. Comput. Vis..

[27] Lin T.Y., Maire M., Belongie S., Hays J., Perona P., Ramanan D., Dollár P., Zitnick C.L. (2014). Microsoft coco: Common objects in context. Proceedings of the Computer Vision–ECCV 2014: 13th European Conference.

[28] Chen K., Wang J., Pang J., Cao Y., Xiong Y., Li X., Sun S., Feng W., Liu Z., Xu J. (2019). MMDetection: Open mmlab detection toolbox and benchmark. arXiv.

[29] Fan Z., Ma Y., Li Z., Sun J. Generalized few-shot object detection without forgetting. Proceedings of the IEEE/CVF Conference on Computer Vision and Pattern Recognition.

[30] Han G., Ma J., Huang S., Chen L., Chang S.F. (2022). Few-shot object detection with fully cross-transformer. arXiv.

[31] Xiao Y., Lepetit V., Marlet R. (2022). Few-shot object detection and viewpoint estimation for objects in the wild. IEEE Trans. Pattern Anal. Mach. Intell..

[32] Hu H., Bai S., Li A., Cui J., Wang L. Dense relation distillation with context-aware aggregation for few-shot object detection. Proceedings of the IEEE/CVF Conference on Computer Vision and Pattern Recognition.

[33] Wu A., Han Y., Zhu L., Yang Y. Universal-Prototype Enhancing for Few-Shot Object Detection. Proceedings of the International Conference on Computer Vision.

[34] Zhu C., Chen F., Ahmed U., Savvides M. Semantic relation reasoning for shot-stable few-shot object detection. Proceedings of the IEEE/CVF Conference on Computer Vision and Pattern Recognition.

[35] Li Y., Zhu H., Cheng Y., Wang W., Teo C.S., Xiang C., Vadakkepat P., Lee T.H. Few-Shot Object Detection via Classification Refinement and Distractor Retreatment. Proceedings of the 2021 IEEE/CVF Conference on Computer Vision and Pattern Recognition (CVPR).

[36] Cao Y., Wang J., Jin Y., Wu T., Chen K., Liu Z., Lin D. (2021). Few-shot object detection via association and discrimination. Adv. Neural Inf. Process. Syst..

[37] Li B., Yang B., Liu C., Liu F., Ji R., Ye Q. Beyond max-margin: Class margin equilibrium for few-shot object detection. Proceedings of the IEEE/CVF Conference on Computer Vision and Pattern Recognition.

